# Correlates of social support on report of probable common mental disorders in Zimbabwean informal caregivers of patients with stroke: a cross-sectional survey

**DOI:** 10.1186/s13104-019-4551-2

**Published:** 2019-08-16

**Authors:** Phillipa Marima, Ropafadzo Gunduza, Debra Machando, Jermaine M. Dambi

**Affiliations:** 1 Department of Rehabilitation, University of Zimbabwe, College of Health Sciences, P.O Box A178, Avondale, Harare, Zimbabwe; 2 Ronelle Isaacs Physiotherapists, 91 Rhino Street, Windhoek, Namibia; 3 Department of Psychiatry, University of Zimbabwe, College of Health Sciences, P.O Box A178, Avondale, Harare, Zimbabwe; 4 Department of Psychology, University of Cape Town, Rondebosch, Cape Town, 7701 South Africa; 5 School of Health and Rehabilitation Sciences, Faculty of Health Sciences, University of Cape Town Observatory, Cape Town, 7700 South Africa

**Keywords:** Stroke, Informal caregivers, Social support, Mental wellbeing, Zimbabwe

## Abstract

**Objective:**

Stroke is a major global public health burden. Unfortunately, stroke invariably leads to functional limitations, consequently, most stroke survivors are hugely dependent on family members/informal caregivers in carrying out essential daily activities. The increased demands of caregiving negatively impact caregivers’ mental health. Nevertheless, caregivers who receive an adequate amount of social support are likely to adjust better to the caregiving role. We sought to determine the impact of social support on the mental wellbeing of 71 caregivers of patients with stroke in Zimbabwe, a low-resourced country.

**Results:**

The mean caregiver age was 41.5 (SD 13.8) years. Patients had a mean age of 65.2 (SD 15.3) years with most being functionally dependent (93.2%). 45.1% of the caregivers showed excessive psychiatric morbidity. The mean Multidimensional Scale of Perceived Social Support (MSPSS) score was 44 (SD 9.4), denoting high levels of social support. Caregivers who received an adequate amount of social support were likely to report of lower psychiatric morbidity (Rho = − 0.285, p = 0.016). Furthermore, caregiver who were; poorer, were caring for more functionally-dependent patients, and did not receive additional assistance were likely to report of poor mental health functioning. There is therefore a strong need to implement context-specific caregivers wellness programs.

**Electronic supplementary material:**

The online version of this article (10.1186/s13104-019-4551-2) contains supplementary material, which is available to authorized users.

## Introduction

Globally, fifteen million people suffer from stroke yearly, and of these, five million are permanently disabled [1]. Although the exact incidence of stroke in Africa is unknown, evidence from systematic reviews points to a significantly increased burden over the past two decades [2, 3]. High rates of HIV infection, rapid urbanization and drastic changes in lifestyles e.g. proliferation of unhealthy diets, increased tobacco and alcohol usage, and increased physical inactivity further compounds the burden of non-communicable diseases such as stroke in low-resourced settings [2–6]. Unfortunately, stroke invariably leads to functional limitations, consequently, most stroke survivors are hugely dependent on family members/informal caregivers in carrying out essential daily activities such as feeding, bathing and dressing [7, 8].

Regrettably, the increased demands of caregiving negatively impact caregivers’ mental health [9–11]. For example; a meta-analysis revealed that depression is endemic in caregivers of stroke patients, yielding a pooled prevalence of 40.2% (95% confidence interval 30.1–51.1%) [12]. Patient characteristics (e.g. severity of stroke and functional limitations), caregiver characteristics (e.g. physical health status, self-efficacy), and contextual/environmental factors (e.g. amount of social support available), influences caregivers’ mental health functioning [8, 10–12]. Positive coping strategies like seeking social support have been proven to lessen the distress associated with caregiving resulting in better mental health outcomes in caregivers of patients with stroke [13, 14]. Social support can be defined as the amount of assistance one gets through interactions with other people. The support can be emotional (e.g. empathy), tangible (practical assistance) or informational (e.g. advice) [15–18]. However, there is a paucity of evidence published information on the buffering effect of social support on the levels of psychiatric morbidity in caregivers residing in low resource settings such as Zimbabwe. The present study sought to fill the gap by profiling the mental health of Zimbabwean caregivers of stroke survivors.

## Main text

### Study design, research setting and participants

Data were collected cross-sectionally from caregivers of stroke patients receiving outpatient rehabilitation care at Chitungwiza Central Hospital (CCH), Parirenyatwa Group of Hospitals (PGH) and St Giles Medical Rehabilitation Centre (SGMRC). CCH and PGH are two of the five largest, public tertiary hospitals in Zimbabwe. SGMRC is a private medical rehabilitation centre located in Harare. An almost similar study yielded a 76% prevalence of common mental disorders (CMDs) in Indian caregivers of patients with stroke [19]. Assuming the following parameters; p_0_ = 0.76, p_1_ = 0.8, α = 0.05 and β = 0.90, the minimum sample size was 70. We recruited; adult (≥ 18 years), primary, unpaid caregivers who voluntarily provided written consent for participation. Caregivers who were suffering from a chronic condition (e.g. HIV/AIDS), had a history of mental illness prior to current caregiving role that led to seeking medical help, and were taking care of another patient with a chronic condition at home were similarly excluded from the study as these contextual factors were potential covariates. Further, we only recruited caregivers proficient in either English or Shona (a Zimbabwean native language), the study outcomes were only available and validated in these languages.

### Study instruments

An ad-hoc sociodemographic questionnaire was designed to extract potential covariates (patient and caregiver characteristics). Psychiatric morbidity and social support were measured using the Shona Symptoms Questionnaire (SSQ) and the Multidimensional Scale of Perceived Social Support (MSPSS) respectively. The SSQ is a 14-item, self-administered, and psychometrically robust screening tool. A reported experience of any of the enlisted 14 symptoms in the past fortnight is rated/scored as 1 with no symptom being scored a 0 giving a cumulative total of 14; scores ≥ 8 are indicative of a high risk of psychiatric morbidity [20, 21]. The MSPSS is psychometrically-robust and extensively used, 12-item social support measure [22, 23]. It serves to measure an individual’s amount of perceived social support received from family, friends and significant others [22]. The Shona version of the MSPSS was used for the current study. Responses on the MSPSS-Shona version are rated on a five-point Likert-type scale ranging from 1 (‘strongly disagree’) to 5 (‘strongly agree’). The scores are interpreted as, the higher scores indicate higher levels of social support [22, 24].

### Procedure

Approval to carry out the study was granted by study sites institutional review boards, and by the Joint Research Ethical Committee for the University of Zimbabwe, College of Health Sciences (JREC Ref: 380/17). The principal investigator (PI) approached prospective participants in the treatment waiting areas. The study aims were briefly explained, thereafter, consenting participants voluntarily completed the study questionnaires. The questionnaires were self-administered, nevertheless, the PI was available to assist the participants whenever necessary. Questionnaires were completed and collected on the same day.

### Data analysis and management

Data were entered and cleaned in Microsoft Excel, and thereafter exported to SPSS (Version 21) for analysis. Descriptive statistics (frequencies, medians and means) were used to describe participants’ demographics and spread of responses on the MSPSS and SSQ. Data were firstly checked for normality using the Shapiro–Wilk test before deciding on the appropriate statistical tests. Consequently, the spearman correlation co-efficient was used to determine the relationship between social support and psychiatric morbidity. Chi square test and student t-test were used to analyse the associations between categorical variables (e.g. gender) and numeric variables (e.g. age) with summative (numeric) scores on the MSPSS and SSQ respectively.

## Results

The mean caregiver age was 41.5 (SD 13.8) years. Most of the caregivers were; females (70.4%), married (56.3%), children to the stroke survivors (52.1%), attained at least secondary education (90.1%), received assistance in caregiving (76.1%), had provided care for at least 4 months, and over 80% of carers reported of inadequate finances. Patients had a mean age of 65.2 (SD 15.3) years, and most of them; were females (53.5%), married (56.3%), suffered from left-sided stroke (52.1%), had lived with the condition for at least 6 months, required assistance in functional activities (93.2%), and hypertension was most common comorbid condition (80.3%). 45.1% of the caregivers showed excessive psychiatric morbidity, the median SSQ score was 7 (IQR: 3–9) (Table 1). Additionally, caregivers received the least and greatest amount of social support from family and friends respectively. The mean MSPSS score was 44 (SD 9.4), denoting high levels of social support. See Additional files 1 and 2 for frequencies of reported problems on the MSPSS and SSQ respectively. Caregivers who received an adequate amount of social support were likely to report of lower psychiatric morbidity (Rho = − 0.285, p = 0.016). Furthermore; caregivers with lower levels of income, were caring for patients with greater functional limitations, and did not receive additional assistance were likely to report of poor mental health functioning (Table 2).

**Table 1:** Participants descriptive statistics, N = 142

Variable	Attribute	Patients, n (%)	Caregivers, n (%)
Gender	Male	33 (46.5)	21 (29.6)
	Female	38 (53.5)	50 (70.4)
Age^a^	Mean (SD)	65.2 (15.3)	41.5 (13.8)
Marital status	Single	4 (5.6)	21 (29.6)
	Married	40 (56.3)	40 (56.3)
	Divorced	4 (5.6)	3 (4.2)
	Widowed	23 (32.4)	7 (9.9)
Side of the body affected	Right side	34 (47.9)	
	Left side	37 (52.1)	
Duration of stroke^a^	Median [Q_1_–Q_3_]	6 (2–16)	
Patient’s level of function	Needs little assistance	2 (2.8)	
	Needs moderate assistance	36 (50.7)	
	Needs maximal assistance	33 (46.5)	
Co-morbidities	Hypertension	57 (80.3)	
	Diabetes	13 (18.3)	
	Arthritis	5 (7.0)	
	Other	7 (15.9)	
Caregiver relationship to the patient	Child	37 (52.1)	
	Spouse	13 (18.3)	
	Sibling	3 (4.2)	
	Parent	2 (2.8)	
	Other relatives	16 (22.5)	
Caregiver educational level	Primary		7 (9.9)
	Secondary		36 (50.7)
	Tertiary		28 (39.4)
Caregiver perceived level of income	Very inadequate		13 (18.3)
	Inadequate		16 (22.5)
	Neutral		29 (40.8)
	Adequate		13 (18.3)
Caregiver received assistance in	Yes		54 (76.1)
caregiving	No		17 (23.9)
Duration of caregiving in months	Median (IQR)		4 (2–13)
Social support (MSPSS) scores^a^	Family [mean (SD)]	4.0 (SD 0.9)	
	Friends [mean (SD)]	3.8 (SD 1.0)	
	Significant other [mean (SD)]	3.2 (SD 1.0)	
	Summative score [mean (SD)]	44 (SD 9.4)	
Psychiatric morbidity (SSQ) scores^a^	SSQ scores ≥ 8 [n (%)]	32 (45.1)	
	Summative score: median [Q_1_–Q_3_]	7.0 [3–9]	

^a^Data not in the n (%) format

**Table 2:** Factors influencing caregivers’ mental health, N = 71

Determinant	SSQ			MSPSS		Interpretation
	Statistic	p-value	Interpretation	Statistic	p-value	
Caregiver’s perceived level of income	*X*^2^ (df = 3) = 12.1	0.007*	Caregivers who reported very inadequate income had the greatest psychiatric morbidity	*X*^2^ (df = 3) = 3.00	0.390	n/a
Patient’s level of function	*X*^2^ (df = 2) = 9.30	0.01*	Caregivers looking after patients who needed maximum assistance had greater levels of psychiatric morbidity	*X*^2^ (df = 2) = 3.80	0.150	n/a
Caregiver received assistance in caregiving	t (df = 69) = − 2.06	0.043*	Caregivers who did not receive any assistance reported the greatest psychiatric morbidity	t (df = 69) = 2.91	0.005*	Caregivers who received assistance had greater social support levels

* Flagged associations were statistically significant

## Discussion

The key findings from the current study were that informal caregivers of patients with stroke were at risk of common mental disorders, and that caregivers who received an adequate amount of social support were likely to exhibit better mental health. Our findings are consistent with previous studies [25, 26]. However, 45.1% of caregivers were at risk of common mental disorders which is ostensibly greater than the 13% lifetime prevalence of common mental disorders in the general population [12, 27]. Nevertheless, the study outcomes are congruent with previous studies on caregivers’ mental health [9–12]. The decreased mental health could be attributable to the cumulative effects of the increased demands of caring for a patient with stroke [10, 11, 25]. For instance, most stroke survivors (93.2%) were functionally dependant on caregivers and this was likely to result in increased physical burden ultimately leading to greater risk of psychiatric morbidity [10]. Further, the median duration of caregiving was 4 months (IQR: 2–13) implying that most participants were still in the acute to sub-acute phases of stroke recovery, a period when the patient is much reliant on the caregiver [25]. Physical health problems such as chronic fatigue, sleep deprivation, pain (particularly shoulder and low back pain) are especially prevalent in caregivers from low-resourced settings [28–30]. The physical burden is further compounded by a lack of appropriate aid and appliances, and ergonomic training resulting in the utilisation of poor lifting and transfer skills [28, 30]. Poor physical health is unfortunately linked to poor mental health outcomes [31–33].

Previous systematic reviews have shown that the cumulative demands of caregiving leads to poor psychological outcomes including; depression, anxiety, post-traumatic stress disorders, amongst others [10, 12]. In this study, the most endorsed items were; insomnia, feeling overwhelmed, thinking too deeply and feeling run down, which is symptomatic of depression [34]. Further, only 18.3% of carers reported of adequate finances, and having lower income was associated with greater risk of psychiatric morbidity. The added financial expenditure and loss of income-generation opportunities exacerbates the economic burden of caregiving thereby creating a vicious cycle of poverty which unfortunately exacerbates the risk of psychiatric morbidity [10, 35]. Furthermore, most of the participants had left-sided stroke which is associated with communication problems [36–38]. Speech problems are one of greatest predictors to poor mental health function in both patients and carers [12, 38, 39]. For example, systematic reviews have demonstrated that patients with communication problems are likely to exhibit poorer HRQoL, are at an elevated risk of depression, and poorer community integration and participation [36, 39]. Poor mental health outcomes in patients secondary to communication problems unfortunately have a negative carryover effects on the caregivers [36, 39, 40]. The communication burden is further exacerbated by the lack of speech and language pathologists in low-resourced settings such as Zimbabwe predisposing both stroke survivors and caregivers to an increased risk of psychiatric morbidity.

Participants in the present study reported high levels of SS and outcomes also support the buffering effects of social support on psychiatric morbidity [13, 14, 25, 41] i.e. caregivers who received an adequate amount of social support had optimal mental health. In this study, caregivers who received some assistance in caring out activities of daily living, a form of instrumental social support [15–18], reported the least psychiatric morbidity. Further, caregivers received the greatest support from the family. This is unsurprising as in the African context as in other collectivistic societies, caring for a patient with a health condition is envisaged as an obligatory, collective family effort [14, 25, 41]. Given that the burden of caring is multifaceted i.e. it can affect caregivers physically, psychologically, emotionally, socially and financially [10, 12], having a supportive social network is indispensable [14, 25, 41, 42]. More so, given the shift towards early discharge and community-based rehabilitation of stroke survivors, the family unit is evolving as an essential component of the rehabilitation process [41, 42]. Consequently, there is greater transfer of the rehabilitation processes to family/informal carers which predisposes caregivers to common mental disorders further underscoring the need for social support [14, 41]. More importantly, a strong support network also enhances caregivers’ adjustment to the caregiving role, and patients’ participation thereby optimising functional recovery, and community participation and reintegration [13, 25].

## Conclusion

Our findings indicate that caregivers of patients with stroke were at risk of psychiatric morbidity, and that caregivers who received an adequate amount of social support were likely to exhibit better mental health. Furthermore, caregivers with lower levels of income, were caring for patients with greater functional limitations, and did not receive additional assistance were likely to report of poorer mental health functioning. There is need to implement caregivers’ wellness programs to improve outcomes in both patients (functional and mental health) and caregivers (mental health and overall well-being). Trained caregivers are likely better adjustment to the demands of caring for a patient with stroke.

## Limitations

Study outcomes need to be interpreted with precautions given the following methodological limitations:

- Data were collected cross-sectionally, causality cannot be inferred.
- Participants were conveniently selected, ideally, we should have randomly selected. Randomization was not possible given the small volumes of patients presenting at the research sites during the study duration.
- Employment of convenience sampling may limit the study’s external validity.
- A lack of a comparison group may potentially limit the study’s external validity. It would have been ideal to compare caregivers’ outcomes with a matched, normative sample.
- Participants’ health status was self-reported, it was therefore difficult to apply a strict/verifiable selection criterion.

## Additional files

**Additional file 1.** Frequencies of responses on the MSPSS, N = 71. Table denotes frequencies of responses on the MSPSS, a 12-item social support outcome measure. Responses are rated on a five-point Likert scale, ranging from “strongly disagree = 1” to “strongly agree = 5”.

**Additional file 2.** Frequencies of responses on the SSQ, N = 71. Table denotes frequencies of responses on the SSQ, a 14-item, binary common mental disorders (CMDs) screen. Respondents indicate if they had experienced any of the enlisted symptoms in the last seven days. A yes response is scored as “one” and no as “zero”, a score ≥ 8 is indicative of risk of CMDs.

## Data Availability

The datasets used and/or analysed during the current study are available from the corresponding author on reasonable request.

## Abbreviations

AIDS: acquired immune deficiency syndrome
HIV: human immunodeficiency virus
IQR: interquartile range
MSPSS: Multidimensional Scale of Perceived Social Support
SSQ: Shona Symptoms Questionnaire
