# Supplementary material for: Correlates of social support on report of probable common mental disorders in Zimbabwean informal caregivers of patients with stroke: a cross-sectional survey
Source: BMC Res Notes. 2019 Aug 16;12:514. doi: 10.1186/s13104-019-4551-2 (PMC6697905; doi:10.1186/s13104-019-4551-2)
Supplement: Supplementary file 1 — Additional file 1. Frequencies of responses on the MSPSS, N = 71. Table denotes frequencies of responses on the MSPSS, a 12-item social support outcome measure. Responses are rated on a five-point Likert scale, ranging from “strongly disagree = 1” to “strongly agree = 5”. [file 13104_2019_4551_MOESM1_ESM.docx]

**Additional File 1: Frequencies of responses on the MSPSS, N=71**

| **Item** | **Response** | | | | |
| --- | --- | --- | --- | --- | --- |
|  | **Strongly Disagree, n (%)** | **Disagree,**  **n (%)** | **Neutral,**  **n (%)** | **Agree,**  **n (%)** | **Strongly Agree,**  **n (%)** |
| 1. There is a special person who is around when I am in need | 6(8.5) | 5(7.0) | 9(12.7) | 32(45.1) | 19(26.8) |
| 1. There is a special person with whom I can share joys and sorrows | 5(7.0) | 6(8.5) | 7(9.9) | 29(40.8) | 24(33.8) |
| 1. My family tries to help me | 49(5.6) | 3(4.2) | 11(15.5) | 25(35.2) | 28(39.4) |
| 1. I get the emotional help & support I need from my family | 3(4.2) | 6(8.5) | 12(16.9) | 29(40.8) | 21(29.6) |
| 1. I have a special person who is a real source of comfort to me | 6(8.5) | 7(9.9) | 13(18.3) | 21(29.6) | 24(33.8) |
| 1. My friends try to help me | 9(12.7) | 8(11.3) | 14(19.7) | 31(43.7) | 9(12.7) |
| 1. I can count on my friends when things go wrong | 17(23.9) | 12(16.9) | 17(23.9) | 18(25.4) | 7(9.9) |
| 1. I can talk about my problem with my family | 3(4.2) | 1(1.4) | 11(15.5) | 29(40.8) | 27(38.0) |
| 1. I have friends with whom I can share my joys and sorrows | 7(9.9) | 7(9.9) | 11(15.4) | 32(45.1) | 14(19.7) |
| 1. There is a special person in my life who cares about my feelings | 5(7.0) | 5(7.0) | 8(11.3) | 31(43.7) | 22(31.0) |
| 1. My family is willing to help me make decisions | 3(4.2) | 2(2.8) | 8(11.3) | 31(43.7) | 27(38.0) |
| 1. I can talk about problems with my friends | 10(14.1) | 9(12.7) | 18(25.4) | 24(33.8) | 10(14.1) |
