# Supplementary material for: Correlates of social support on report of probable common mental disorders in Zimbabwean informal caregivers of patients with stroke: a cross-sectional survey
Source: BMC Res Notes. 2019 Aug 16;12:514. doi: 10.1186/s13104-019-4551-2 (PMC6697905; doi:10.1186/s13104-019-4551-2)
Supplement: Supplementary file 2 — Additional file 2. Frequencies of responses on the SSQ, N = 71. Table denotes frequencies of responses on the SSQ, a 14-item, binary common mental disorders (CMDs) screen. Respondents indicate if they had experienced any of the enlisted symptoms in the last seven days. A yes response is scored as “one” and no as “zero”, a score ≥ 8 is indicative of risk of CMDs. [file 13104_2019_4551_MOESM2_ESM.docx]

**Additional File 2: Frequencies of responses on the SSQ, N=71**

| **Item** | **Response** | |
| --- | --- | --- |
|  | **Yes, n (%)** | **No, n (%)** |
| 1. Thinking too deeply | 51 (71.8) | 20 (28.2) |
| 1. Failing to concentrate | 33 (46.5) | 38 (53.5) |
| 1. Get easily annoyed | 36 (50.7) | 35 (49.3) |
| 1. Nightmares | 22 (31.0) | 49 (69.0) |
| 1. Hallucinations | 4 (5.6) | 67 (94.4) |
| 1. Stomach aching | 12 (16.9) | 59 (83.1) |
| 1. Frightened by trivial things | 12 (16.9) | 59 (83.1) |
| 1. Fail to or lose sleep | 45 (63.4) | 26 (36.6) |
| 1. Cried or wanted to cry | 43 (60.6) | 28 (39.4) |
| 1. Feel run down | 49 (69.0) | 22 (31.0) |
| 1. Feel like committing suicide | 5 (7.0) | 66 (93.0) |
| 1. Generally unhappy | 33 (46.5) | 38 (53.5) |
| 1. Work lagging behind | 41 (57.7) | 30 (42.3) |
| 1. Problems in making decisions | 33 (46.5) | 38 (53.5) |
